# Striatal Activity and Reward Relativity: Neural Signals Encoding Dynamic Outcome Valuation

**DOI:** 10.1523/ENEURO.0022-16.2016

**Published:** 2016-11-01

**Authors:** Emily S. Webber, David E. Mankin, Howard C. Cromwell

**Affiliations:** 1 Department of Psychology, Bowling Green State University, Bowling Green, Ohio 43403; 2 J.P. Scott Center for Neuroscience, Mind & Behavior, Bowling Green State University, Bowling Green, Ohio 43403

**Keywords:** electrophysiology, goal-directed action, incentive contrast, motivation, nucleus accumbens, single unit recording

## Abstract

The striatum is a key brain region involved in reward processing. Striatal activity has been linked to encoding reward magnitude and integrating diverse reward outcome information. Recent work has supported the involvement of striatum in the valuation of outcomes. The present work extends this idea by examining striatal activity during dynamic shifts in value that include different levels and directions of magnitude disparity. A novel task was used to produce diverse relative reward effects on a chain of instrumental action. Rats (*Rattus norvegicus*) were trained to respond to cues associated with specific outcomes varying by food pellet magnitude. Animals were exposed to single-outcome sessions followed by mixed-outcome sessions, and neural activity was compared among identical outcome trials from the different behavioral contexts. Results recording striatal activity show that neural responses to different task elements reflect incentive contrast as well as other relative effects that involve generalization between outcomes or possible influences of outcome variety. The activity that was most prevalent was linked to food consumption and post-food consumption periods. Relative encoding was sensitive to magnitude disparity. A within-session analysis showed strong contrast effects that were dependent upon the outcome received in the immediately preceding trial. Significantly higher numbers of responses were found in ventral striatum linked to relative outcome effects. Our results support the idea that relative value can incorporate diverse relationships, including comparisons from specific individual outcomes to general behavioral contexts. The striatum contains these diverse relative processes, possibly enabling both a higher information yield concerning value shifts and a greater behavioral flexibility.

## Significance Statement

This study is the initial research directly linking striatal activity to relative incentive contrast processes during instrumental action. The work not only demonstrates how striatal activity can dynamically encode specific outcome value information, but also shows that striatal activity simultaneously encodes value at different levels using different types of information concerning the outcome and related context. The results link the neuroscience of reward to motivational theory that guides key experimental behavioral work on choice, decision-making, and goal-directed action. Recent work has shown that mental illnesses such as schizophrenia and addiction are accompanied by deficits in reward valuation; and the present study provides key insight into neural processes that could be altered, leading to emotional and behavioral impairments in mental disorders.

## Introduction

Stable neural representations have been important components of motivational neuroscience (Hikosaka et al., 2014; Bowman et al., 1996; Ikemoto et al., 2015; Morita and Kawaguchi, 2015) and historically helped strengthen the idea that a hierarchy of incentives can reliably guide behavior and brain function (Young, 1959; Berridge, 2004). For the striatum in particular, there appears to be a set of stable neural activations related to expectancies for reward and particular types or quantities of reward (Hollerman and Schultz, 1998; Hassani et al., 2001; Cromwell and Schultz, 2003; Cromwell, 2010; Nunes et al., 2013).

How long representations of value endure in a stable fashion and to what extent value shifts are encoded by proportional shifts in neural activity are unknown. It is clear that striatal activity does change, and this can rely crucially upon parameters of motivation (Flaherty and Graybiel, 1991; Peters and Büchel, 2010; Louie et al., 2014). Striatal activations linked to predictive cues, delay periods, reward, and expectation depend upon relative value (Cromwell et al., 2005; Kim et al., 2008). One type of relative outcome effect has been observed as a shift in activity from one reward to another following value alteration. This form of activity has been found in orbitofrontal cortex as well as striatum and been proposed to represent preference, independent of other outcome properties (Tremblay and Schultz, 1999; Cromwell et al., 2005). Another type of effect was observed as activity intimately linked to a specific outcome, yet changing dependent upon the incentive value of an alternative outcome (Cromwell et al., 2005). This outcome-linked activity appears to be more related to incentive relativity as a shifting indicator of value connected to a specific outcome. Yet to be explored is the impact of different levels of value shifts in both positive and negative directions on striatal activity, and how influences of value shifting are linked to specific events and actions using a predetermined level of “absolute” value for each outcome.

In order to investigate relative reward effects on neural activity more thoroughly, we examined striatal activity during a paradigm that enables relative and rapid comparisons in the tradition of incentive contrast studies (Crespi, 1942; Blough, 1980). Experimental work on incentive contrast has a rich history and has been invaluable in deciphering components of motivation that integrate to form value-based decisions. It essentially explores the impact of reward value upshifts (positive contrast) and downshifts (negative contrast). A critical component of incentive contrast is that the key comparisons are between identical outcomes (Flaherty, 1996). This absolute value control is often lacking in behavioral and neurophysiological work, and, without its use, it is difficult to delineate relative valuation like contrast from other reward processing components, such as discrimination and preference (Ricker et al., 2016a). The outcomes are experienced as either an “absolute outcome” because of the absence of comparable outcomes or as a “relative outcome” when embedded in a set of alternatives. This comparison enables relative outcome valuation to proceed without fundamentally relying upon discrimination between different outcome parameters (Watanabe et al., 2001). Recently, we have developed a novel behavioral paradigm that contains the necessary characteristics to facilitate reward comparison and allow for tracking neural activity. We obtained diverse relative reward effects on behavior. Positive and negative-contrast effects were obtained as well as other incentive value relations (Webber et al., 2015).

These other diverse relative outcome effects on behavior reflect generalization of incentive value from either the overall context of the reward situation or convergence of specific, distinctive outcomes. An example of general context effects would be variety influences, with possible incentive value shifts depending upon more or less variety within a situation (Webber et al., 2015). Variety effects are defined as enhanced responding when animals are exposed to diverse sets of alternatives compared with exposure to repetitive identical items (Thrailkill et al., 2015). This form of relative encoding mainly disregards single outcomes and primarily focuses on general properties, such as the size of the outcome set or the rate of outcome shifts. Variety effects can involve dishabituation and contrast to reduce sensory-specific satiety, and invigorate food consumption and operant responding for food (Bouton et al., 2013; Thrailkill et al., 2015). Another generalization effect on behavior was obtained that appeared as a transfer of value from one outcome to another (Webber et al., 2015). This process is similar to an effect-labeled induction, which is typically seen as an increase in responding for a less valued relative outcome and the degree of change is directly related to the relative value of the paired alternative higher valued outcome (Segal, 1972; Weatherly et al., 2001). Induction can occur as a connection is made among outcomes, and a similar response is produced to an outcome set. Typically, this behavioral phenomenon occurs when an outcome of higher value dominates the outcome landscape and produces a general invigoration of responding. This “induction” effect contradicts contrast and can lead to a similar level of responding to outcomes that have significantly different value along the incentive hierarchy. Typically induction reduces negative contrast leading to similar responses for all comparable outcomes. (Weatherly et al., 2005).

To investigate striatal participation in relative outcome processing using the behavioral work built upon incentive-contrast studies, we monitored neural activity across several sessions that differ in terms of initial experience (a lower or higher magnitude outcome) and the disparity between outcomes (larger or smaller differences in magnitude). The role of the striatum in terms of incentive contrast could be to form transient value representations that are easily accessible to motor output and influence future motivation to work for an outcome. We explored activity from both dorsal striatum (DS) and ventral striatum (VS) to determine the extent of functional heterogeneity related to different forms of relative reward processing. One functional proposal has the DS as the “actor” importantly involved in functions related to action value encoding (Ito and Doya, 2015a), action sequencing (Cromwell and Berridge, 1996), and stimulus–response habit formation (Yin et al., 2004; Featherstone and McDonald, 2005). These functional attributes could be less dynamic and flexible, enabling performance in well predicted situations and possibly reducing outcome value influences (Yin et al., 2006; Burton et al., 2015). In contrast, VS has been linked more to the processing of reward value outcome in diverse behavioral paradigms (Cai et al., 2011; Ricker et al., 2016b) The VS has been labeled the “critic” because of the potential for dynamic value assignment (O’Doherty et al., 2004). Support is mixed for these and other functional dissociations between DS and VS with evidence supporting a distributed or parallel nature to striatal processing (Cromwell et al., 2005; Ito and Doya, 2015b; Smith and Graybiel, 2016). The present work using a new paradigm could improve the ability to determine the extent and type of functional dissociation between these two striatal subregions.

We hypothesize that relative reward encoding will be reflected by shifts in neural activity related to outcome value when the alternative outcome value changes. Specifically, we predicted that when rewards are paired with a lower value outcome, event-related activity will significantly increase as a “neural” positive incentive contrast, and when outcomes are paired with an alternative of higher value, the opponent neural negative contrast will occur. These shifts will be dependent upon the degree of disparity of the value shift. Finally, we predict that neural signals related to relative valuation will be more prominent in the VS compared with the DS, especially surrounding the reward delivery and acquisition because of the putative critic role for VS that incorporates dynamic outcome information. These studies have important implications for merging traditional motivational work with current neurophysiology of reward processing and can lead to novel ways to explore reward deficits in psychopathology.

## Materials and Methods

### Animal subjects

Animals (*n* = 10 male, adult Sprague Dawley rats; *Rattus norvegicus*) were housed individually (cage dimensions, 65 × 24 × 15 cm) and weighed between 220 and 290 g at the start of the experiment. Animal weights were measured for 3 consecutive days to obtain baseline weights, and then animals were food restricted to 87–90% of this baseline weight for the duration of training and testing. Animals were given 5–15 g of food immediately after training or testing to maintain the restricted weight. The colony housing room was on an automatic 12 h light/dark cycle beginning at 8:00 A.M. The temperature of the room was 22^°^C with 40–55% humidity. All procedures of this study were performed in accordance with the *Guide for the Care and Use of Laboratory Animals* and were approved by the Bowling Green State University Institutional Animal Care and Use Committee (Protocol 10-015).

### Behavioral training

**Figure 1.**
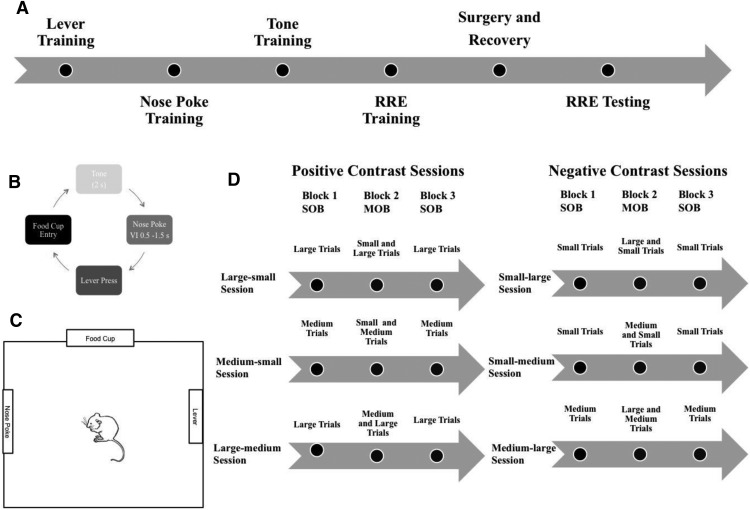
***A***, General timeline for the experimental procedure. ***B***, Diagram of each of the steps involved in the instrumental task. ***C***, Representation of the operant chamber. ***D***, This diagram shows trial types that were presented in each block for each session type. Sessions are grouped by contrast type. Each animal underwent testing in each session type. Session order was presented in a pseudorandom fashion.

Animals were trained in a Plexiglas chamber (31 × 31 × 25 cm) with an operant nosepoke on the left-side wall, a food cup on the middle wall across from the door, and a lever located on the right side of the chamber (Fig. 1). All devices were controlled by computer software and hardware systems (MedPC, Med Associates Inc.). Custom-written programs were used to control input and output signals to each device, including inputs from nose poking, lever-pressing, as well as food cup entry to retrieve food pellets used as the reward outcome (45 mg chocolate-flavored sucrose; BioServ Inc.). Food pellets were dispensed using an output signal sent to a food dispenser connected to the food cup and located outside of the chamber.

Each animal was initially trained to press the lever on a fixed ratio 1 schedule (with one press for three pellets; Fig. 1). Once the animals were consistently responding (30 trials in a 20 min session), they were trained to hold their snout (0.5–1.5 s) in the nosepoke in order for the lever to extend. Finally, after the identical learning criterion (30 trials in a 20 min session), animals were exposed to the tone stimuli that would allow for prediction of the upcoming reward magnitude. Three tone stimuli (2, 4, or 6 kHz) were paired with three different levels of food magnitude (one-, two-, or four-pellet amounts). The tone–outcome pairs were counterbalanced across subjects. The sequence of events in a trial proceeded as follows: (1) tone stimulus followed by (2) nosepoke hold, then (3) lever-extension and lever-press, and (4) food delivery and consumption. Animals were trained using a 2 s tone stimulus duration and experienced single-outcome sessions for each food pellet magnitude until each subject demonstrated significant discrimination for one measure (nosepoke, lever-press, or food cup latency) among the three different magnitude session types (one, two, or four pellets with latencies showing faster responses with four-pellet trials < two-pellet trials < one-pellet trials for trial averages of response latencies).

### Surgery

Once animals obtained the necessary behavioral requirements for discrimination among magnitudes, they were given 3 d *ad libitum* access to food and water in preparation for surgery. Anesthesia was administered using isoflurane initially in an induction chamber (5%) and then using a nosecone (1–3%) with a precision vaporizer (EZ Anesthesia). Buprenorphine (Buprenex, Reckitt Benckiser Healthcare**)** was administered 15–20 min prior to general anesthesia. The scalp was shaved, and the animal was placed into a small animal stereotax (Kopf Instruments) using blunt 45^°^ earbars to stabilize the head position and to prevent any damage to the ear. Sterile eye ointment (Webster) was used to prevent desiccation. A povidone-iodine solution (10% Betadine, Henry Schein, Inc.) was used as an antiseptic treatment for the skin, and lidocaine (0. 2 ml) was injected as a topical analgesic. A longitudinal incision was made, and skin was retracted over the skull. To ensure precise electrode implantation, the bone landmarks (lambda and bregma) were leveled, and skull screws were inserted (two anterior and two posterior) following bilateral craniotomies for left and right hemisphere electrode implantation.

### Electrode implantation and recovery

Microelectrodes were custom-ordered arrays (Neuro Biological Laboratories) of two 2 × 4 electrode configurations. Electrodes were made with Teflon-coated stainless steel (50 µm diameter with 250 µm distance) using connectors (Omnetics Connector Corporation) with solder pins, and the wires were attached to connector pins. Arrays were lowered down (1 mm/min) into the dorsal [anterior (A), +0.7–1.2; mediolateral (M), ±2.9; dorsoventral (DV), −4.7 mm relative to bregma; Paxinos and Watson, 1985] and ventral striatum (A, 0.7–1.2; M, ±0.7–1.0; DV, −7.5 mm). Once electrodes reached target location, cyanoacrylate (World Precision Instruments) and methyl methacrylate (Sigma-Aldrich) were used to cover the craniotomy, and the skull and skull screws. This preparation secured the electrode array as a fixed chronic implant for the duration of testing. Animals were allowed to recover for 7 d postsurgery prior to behavioral retraining and testing. Antibiotic ointment (Neosporin, Johnson & Johnson) was used around the wound site, and buprenorphine (Buprenex 0.05 mg/kg) was administered every 6 h for 2 d. Oral enrofloxacin (Baytril, Bayer Healthcare) in sweetened grape juice was provided for 7 d postsurgery (500 mg in 400 ml of fluid). Baseline weights were remeasured on days 5–7 during recovery and then food restriction restarted to drop the animal weights to 87–90% of the new baseline weights.

### Relative reward effect testing

Animals were retrained for 3 d with daily sessions of each reward magnitude type. Each subject had to meet the criterion for significant discrimination for the response latencies among the different reward magnitudes (four-pellet response < two-pellet response < one-pellet response signifying faster response times for at least a single behavioral measure) in order to proceed to relative reward testing. Unit recording (described below) was completed during each day of relative reward testing. In order to obtain potential positive or negative-contrast effects, we used six different sets of three session types per day. Each subject was exposed to each of the six “contrast” session sets in a counterbalanced order. Each daily contrast test was composed of three sessions with an intersession interval of 45 min. The three sessions included the following: (1) an initial single-outcome session with 40 trials of one reward magnitude (one, two, or four pellets); followed by (2) a mixed session with 20 trials of two different pellet magnitudes (e.g., one- and two-pellet trials); followed by (3) a second single session with 40 trials of the same outcome as session 1. This third session was used as a control to determine the impact of satiety on motivated behavior. If behavior was altered between the initial and final single-outcome sessions, then it would signal the possibility of either a satiety effect with slowed response times or a general sensitization effect from repeated experience with the task contingency. The daily positive-contrast sessions included (1) a large-magnitude single session and a large–small-magnitude mixed session, (2) a medium-magnitude single session and a medium–small-magnitude mixed session, or (3) a large-magnitude single session and a large–medium-magnitude mixed session. Since pellet numbers overall were higher for this final contrast session, trial numbers were reduced from 40 to 20 for each session. The negative-contrast sessions included the following: (1) a small-magnitude single session and a small–large-magnitude session; (2) a medium-magnitude single session and the medium–large-magnitude mixed session; and the (3) a small-magnitude single session and the small–medium-magnitude mixed session.

### Neural data acquisition

All units were obtained from these six daily sessions for each subject. For each relative reward test-recording day, neural signals were transferred to the acquisition system (Multichannel Acquisition System, Plexon Inc.) using a high-impedance headstage and a cable (16 channel with ground reference and 1× gain). The cabling wire was surrounded by plastic wrap, and was flexible to allow for freedom of movement in the operant chamber. Signals were amplified (1000–20000×) and collected using a window or threshold discrimination method. All waveforms that met threshold criteria were timestamped and stored at 40 Hz. All waveforms were then examined and analyzed off-line (Offline Sorter, Plexon Inc.). We used template matching and principle component analysis to remove non-template-matching units and artifacts, and delimiting clusters to produce more conservative units for analysis. Each single unit had the following: (1) an amplitude peak to peak of at least 90 µV; (2) at least a 4:1 signal-to-noise ratio; (3) <5% of the interspike intervals could be <3 ms; and (4) the mean baseline firing rate was <40 Hz. Each unit had to show activity during the session from the initial 1 min baseline recording period and during the final session of the day in order to be included in the relative reward effect analysis. We used these methods in an attempt to ensure a low rate of error in reclassifying any one unit as a novel unit compared to a previous session, but could have a margin of error that led to an oversampling of unit numbers arising from classifying a unit distinct and novel when it was a unit retained from a previous recording session.

### Unit and response classification

We divided the analysis into different levels that focused on these categories: (1) event relationship; (2) relative reward effect; (3) direction of activity change; and (4) striatal neuronal subpopulation. These diverse categories were applied to subsets of units obtained from either the dorsal or ventral striatum. For the event relationships, we used baseline unit activity obtained from three consecutive 60 ms bins prior to tone onset. Since the tone presentations were variable and unpredictable, this time period represents a period of baseline activation unassociated with other events. To be classified as an event-related response unit activity had to be statistically significant (see Data analysis) above baseline for at least three consecutive bins (60 ms each). We partitioned the trials into the following events: (1) tone-related activity was examined 2 s following tone onset; (2) nosepoke activation was measured starting 120 ms prior to nosepoke; (3) lever-press activation began at the time of lever-press; and (4) food cup activation was measured at the time of food cup entry. The duration of activation was terminated when three consecutive bins of nonsignificant change in unit activity occurred (for similar event-related definitions, see Cromwell and Woodward, 2006; Mears et al., 2006). Event-related significant activations were examined for each of the three sessions on each of the 6 d of testing. The relative reward effect analysis examined each of the event-related activations and categorized them into (1) positive contrast, (2) negative contrast, (3) mixed session, or (4) nonrelative reward effect-related activations. To examine these relative reward effect activations, we completed statistical analysis among the three daily sessions. It was not necessary to retain the same single units across days in order to complete the basic relative reward analysis. Single-unit activity was examined from the same trial type (e.g., identical pellet magnitude) among the initial single-outcome session, the middle mixed-outcome session, and the final single-outcome session. For positive-contrast analyses, these comparisons were performed when the larger reward was the single-outcome type and the mixed session paired this larger outcome (large or medium outcome) with a smaller outcome (medium or small outcome). Significant differences had to occur between the trials for the larger outcome between the single sessions and mixed session. For the negative-contrast category, the comparisons were completed using the smaller outcome in single sessions and the combinations of the smaller and larger outcomes in the mixed session. Similar significant differences were essential between the two session subtypes. Finally, the mixed-session category included activity that was significantly different from baseline only during the mixed session with combined outcomes or selective activity changes in the mixed session that were inconsistent with the incentive-contrast predictions. The relative reward effect-related activations were further subdivided into direct or inverse coding subtypes. Direct coding refers to responses that showed increases in firing rates as the reward level increased (i.e., higher firing rate for larger rewards). Inverse coding refers to responses that showed inhibition in firing rates as the reward level increased (lower firing rates for larger rewards). Finally, we explored putative neuronal classifications into medium spiny neurons (MSNs) or fast-spiking neurons. We subdivided these relative reward effect responses into these categories based upon spike waveforms (from peak width at half-maximum amplitude and firing rate (MSNs <8 Hz with 150 to 500 µs widths and fast spiking interneurons with >8 Hz and 75–150 µs widths).

### Data analysis

Analysis of neural data was completed using Neuroexplorer (NEX, Plexon), Excel (Microsoft), and MATLAB [MathWorks version 5.4 (RRID:SCR_001622)]. Plots and figures were completed in R [R Project for Statistical Computing (RRID:SCR_001905; http://www.r-project.org)]. We used 60 ms bins for unit analysis, and produced perievent rasters and histograms for neural event timestamps to examine neural event relationships. For statistical analysis we used custom-written MATLAB scripts to convert activity into *z*-scores. The *z*-scores for each unit were averaged, and these averages were used to conduct population analyses: *z* = (average of unit bin – average of baseline bin)/SD of baseline bin). Wilcoxon signed-ranked tests were used to make comparisons within the positive and negative-contrast groups. Nonparametric tests were used for all neural data comparisons because the data violated the assumption of normal distribution. Sidak–Bonferroni corrections were used for neural relative reward effect (RRE) analysis and the critical α level was set to *p* = 0.033. The rationale for correction factor was based on the three consecutive sessions and multiple comparisons across these three repeated measures of behavioral testing. χ^2^ tests were used to examine differences in proportions of responses between categories and target locations.

A set of analyses between days was completed to examine whether or not activity changes were related to the degree of outcome magnitude change across days. A small fraction of the responses was used for this between-day analysis, and the single unit had to match the template parameters stored from one day to the next, meaning that the height and width of the waveform had to be stable. In addition, the unit had to have nonsignificant change or no change in baseline firing and show the same event-related activation. All units were inspected for activity using perievent histograms. This degree of change analysis used units that were recategorized into a one-step or two-step subtypes in order to examine whether the disparity in magnitude between larger and smaller pellet reward impacted the degree of change seen in RRE neural firing. The one-step category contained sessions in which the difference between the two available rewards was small or only one level of difference. These included the medium-small, small-medium, medium-large, and large-medium sessions. The two-step category contained sessions in which the difference between the two available rewards was large, or two levels of difference. These included the small-large and large-small sessions. The percentage change for positive contrast (% change = (100 − (single higher reward/mixed higher reward) × 100)) and for negative contrast (% change = (100 − (single higher reward/mixed higher reward) × 100)) was determined. Paired-samples *t* tests were used to compare the percentage change between one-step and two-step for each behavioral measure. Data did not violate the assumption of normal distribution. The one-step category contained more single units than the two-step category because it contained more session types, and units were randomly removed from the one-step categories to ensure equal sample sizes (nose poke, *n* = 6; lever-press, *n* = 12; food cup, *n* = 16).

Behavioral data were analyzed using nonparametric statistical tests including Friedman’s ANOVA to gain omnibus effects across sessions and Wilcoxon signed-rank tests for between-session comparisons. Data analysis was completed using PASW Statistics (SPSS Inc. version 18), and graphs were made using SigmaPlot (version 12) and R (The R Project Statistical Compiling).

### Microelectrode mapping

**Figure 2.**
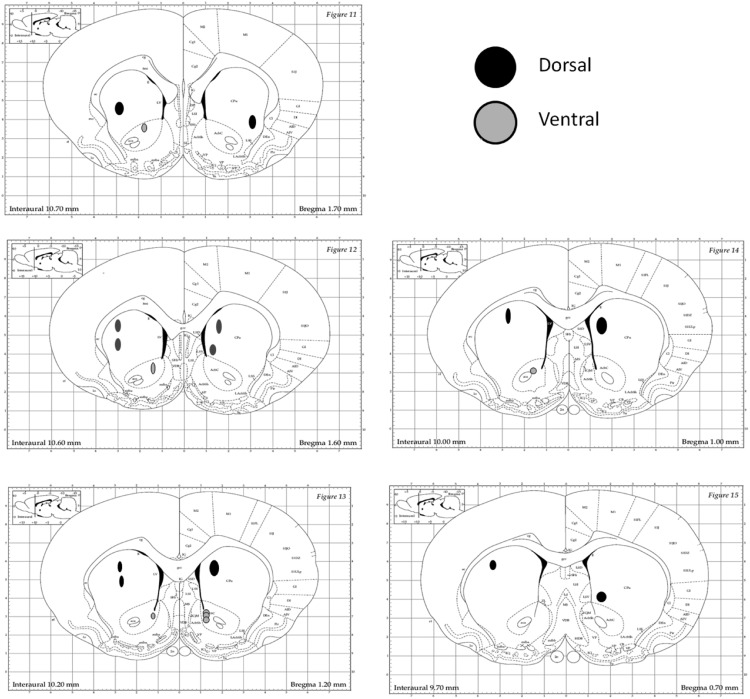
Histological verification of microwire implantation sites in the dorsal and ventral striatum. Coronal sections are displayed from anterior to posterior sections, and wires located in dorsal (black circles) and ventral (gray circles) are shown. Dorsal sites were distributed in all quadrants, including dorsomedial striatum and dorsolateral striatum. All ventral striatal sites were in medial segments, including medial core and shell subregions.

After completion of testing rats were anesthetized using 100 mg/kg (i.p.) pentobarbital. After anesthesia, animals were perfused with a 0.9% saline solution followed by a 10% solution of phosphate-buffered formalin. This was consistent with American Veterinary Medical Association guidelines for the killing of the animals. Just prior to perfusion, 10 mA of current was passed for 15 s through every other microwire of each bundle of the recording microelectrodes to mark their placement with a lesion. After perfusion, brains were removed and stored in perfusion solution for 1 d and then transferred to 30% sucrose/10% formalin solutions for 24 h. The target sections containing the striatum were mounted on glass slides and stained with cresyl-violet. The brain slices were scanned under a digitizing microscope and analyzed to make sure that each microwire was placed in the intended areas of the striatum. A standard rat stereotaxic atlas was used for verifying correct microwire implantation (Paxinos et al., 1985). Microwire sites were identified using a microscope and were mapped onto images from the Brain Atlas of Paxinos et al. (1985; Fig. 2). In order for a lesion area to be confirmed, there must have been a hole present and surrounded by dark purple Nissl bodies, indicating tissue damage. Fiber tracts leading to holes or darkened areas were also considered to be evidence of microwire presence. Two of the lesions appeared to be just ventral to the ventricle, and in these cases a hole was not seen, but clear fiber tracts were seen leading through the ventricle and terminating in a darkened area ventral to the ventricle. Twelve microwire implantation areas were identified (Fig. 2). Six of these areas were found in the dorsal (three left, three right) striatum, and six were found in the ventral (three left, three right) striatum.

## Results

### Behavior during the incentive-contrast sessions

#### Positive-contrast effects

**Figure 3.**
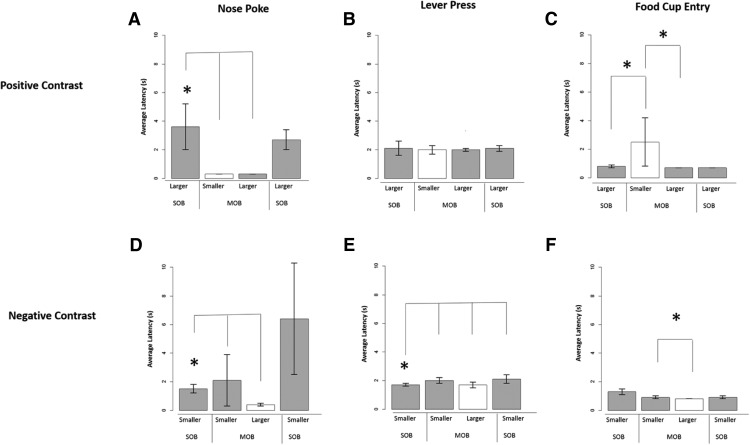
***A–F***, Behavioral response latencies during positive-contrast (***A–C***) and negative-contrast (***D–F***) session types. ***A***, Nose poke: there was a generalized decrease in response latencies during the MOB session when compared with the SOB session. ***B***, Lever-press: the lever-press response did not significantly differ between the single- and mixed-outcome sessions or during the smaller vs larger trials in the mixed session. ***C***, Food cup entry: results showed an increase in food cup entry latencies during the mixed session, as well as a significant discrimination effect during this same session block. ***D***, Nose poke: there was a generalized decrease in response latencies during the mixed session when compared with the single-outcome session. ***E***, Lever-press: animals showed steady increases in latency to lever-press over trial blocks for the smaller reward outcome. ***F***, Food cup entry: results showed significantly longer latencies to enter the food cup during smaller than larger reward trials in the mixed-outcome session. Bars represent averages of normalized data, and error bars denote the SEM for each outcome presentation. Gray bars depict the similar outcome across trial blocks, and white bars depict the alternate outcome.

Six subjects were used for the neural data analysis, and their behavioral data are presented from the relative reward testing sessions. For each of the three positive-contrast sessions, there was a main effect for the nosepoke latency (Fig. 3A; low-high session: χ^2^(3) = 14.9, *p* = 0.002; medium-high session, χ^2^(3) = 14.8, *p* = 0.002; low-medium session, χ^2^(3) = 15.2, *p* = 0.002). In each comparison, animals produced significantly shorter latencies to nosepoke for the larger outcome in the mixed-outcome block when compared with the high-reward trials in the initial single-outcome block (low-high sessions, *z* = −2.25, *p* = 0.02; medium-high session, *z* = −2.201, *p* = 0.02; and low-medium session, *z* = −2.201, *p* =0.02). This result is exactly what was predicted for positive contrast, with faster response times in the mixed session compared with the single-outcome session. As found in previous work (Webber et al., 2015), we also had significantly shorter latencies for the alternative outcome during the mixed session in all three positive-contrast session types [low outcome (vs high), *z* = −2.201, *p* = 0.028; medium outcome (vs high), *z* = −2.19, *p* = 0.02); and medium outcome (vs low), *z* = −2.27, *p* = 0.01]. This latter effect could depend upon outcome generalization within the mixed session, leading to faster responses for each outcome during this testing period. Behavioral responses were also faster for the lever-press and the food cup entry, but not significant between the single and mixed session (Fig. 3B,C; *p* > 0.033). These latter two responses in the chain had shorter latencies compared with the initial instrumental behavior of the nosepoke.

#### Negative-contrast sessions

For two negative-contrast sessions, there was a main effect for the nosepoke latency (Fig. 3D; high-low session: χ^2^(3) = 14.6, *p* = 0.002; high-medium session, χ^2^(3) = 14.800, *p* = 0.002). Animals produced significantly longer latencies to nose poke in the mixed trials compared with single trials for the lower outcome (low outcome, *z* = −2.24, *p* = 0.02; medium outcome, *z* = −2.201, *p* = 0.02). Shorter latencies were obtained for the higher-reward trials [high (vs low), *z* = −2.201, *p* = 0.02 and high (vs medium), *z* = −2.23, *p* = 0.02] in the mixed-outcome block. The lever-press did show negative-contrast effects with a trend of trial type on latency to lever-press during the high-low-contrast series (Fig. 3E; χ^2^(3) = 8.600, *p* = 0.035). Slower lever-press latencies did occur in the mixed middle session (*z* = −2.19, *p* = 0.02) and continued to be slower in the final single-outcome session (*z* = −2.201, *p* = 0.028). Both the nosepoke and lever-press met expectations with slower latencies found for the lower outcome during the mixed session. There was a main effect of trial type on latency to enter the food cup (high-low, χ^2^(3) = 10.400, *p* = 0.015; medium-low, χ^2^(3) = 9.800, *p* = 0.02; and high-medium session, χ^2^(3) = 11.339, *p* = 0.010). The results were opposite to negative-contrast expectations with actual shorter latencies during the mixed session for the lower outcome (Fig. 3F; high-low session, *z* = −2.201, *p* = 0.02; medium-low session, *z* = −2.201, *p* = 0.02; and high-medium session, *z* = −2.201, *p* = 0.02). Similar faster latencies were found for the larger outcome in each mixed session as well [high (vs low), *z* = −2.22, *p* = 0.02; high (vs medium), *z* = −2.201, *p* = 0.02 and medium (vs low), *z* = −2.27, *p* = 0.01].

### Neural data results

A total of 1,113 units were recorded over the six session types. All neural data were obtained from six subjects run on each session type for a total of 36 sessions. A total of 543 units (283 tested during positive comparison tests, 260 tested during negative comparison tests; *p* > 0.05) were recorded from the dorsal striatum (Table 1). The units were divided into the four event-related response subtypes. Tone, nosepoke, lever-press, and food cup responses (383 responses from 330 DS units) ranged from 11% to 32% of the total. The numbers of responses surpasses the number of units because some units had more than a single event-related activation. Most of these combined activations were lever-press–food cup responses (78%), but tone–lever-press (14%) and nosepoke–lever-press (8%) were also found. In the VS, 570 units were obtained and 398 of the units (70%) were responsive to one of the events. The highest proportion of 486 responses (236 tested during positive contrast and 250 during negative contrast) were obtained at the time of food cup entry and pellet retrieval (44%; Table 2), but activations were found at other events as well (Table 2).

**Table 1:**
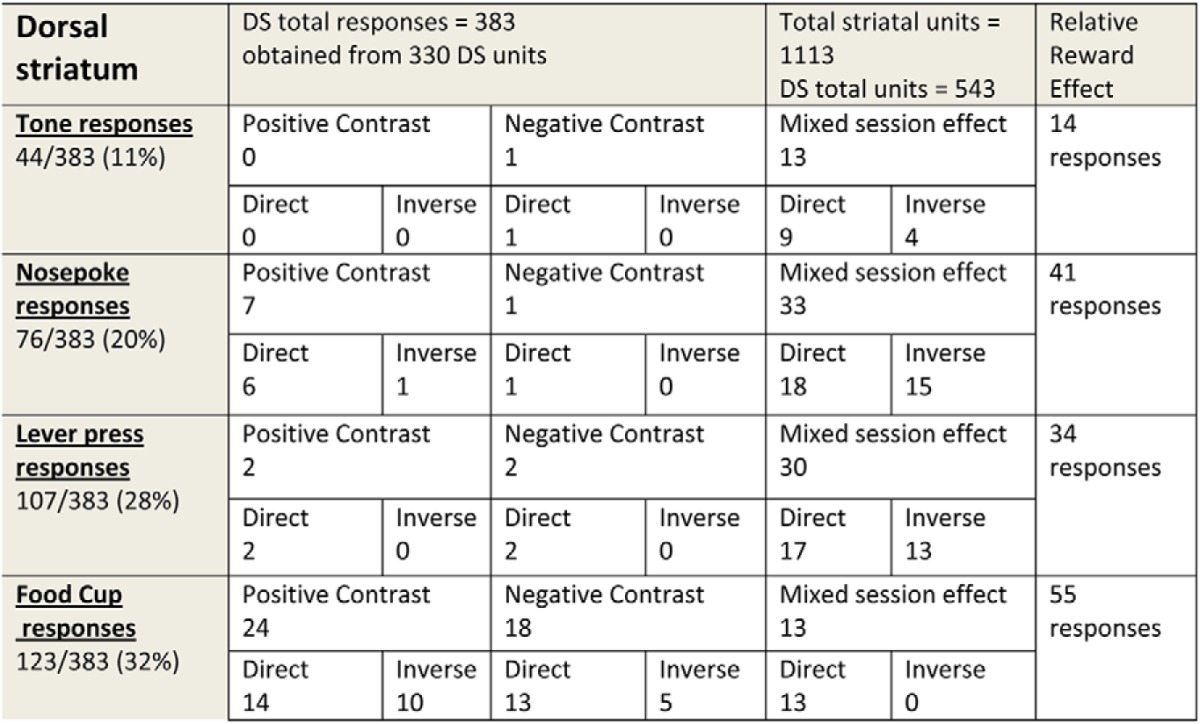
Single units and event responses from dorsal striatum

Single units and responses from the DS classified into event type and divided by one of the three relative reward effects: positive contrast, negative contrast, or mixed session effects. See the text for definitions for each of these relative reward effects. Each of these subtypes of responses is divided by the direction of activity change related to reward magnitude.

**Table 2:**
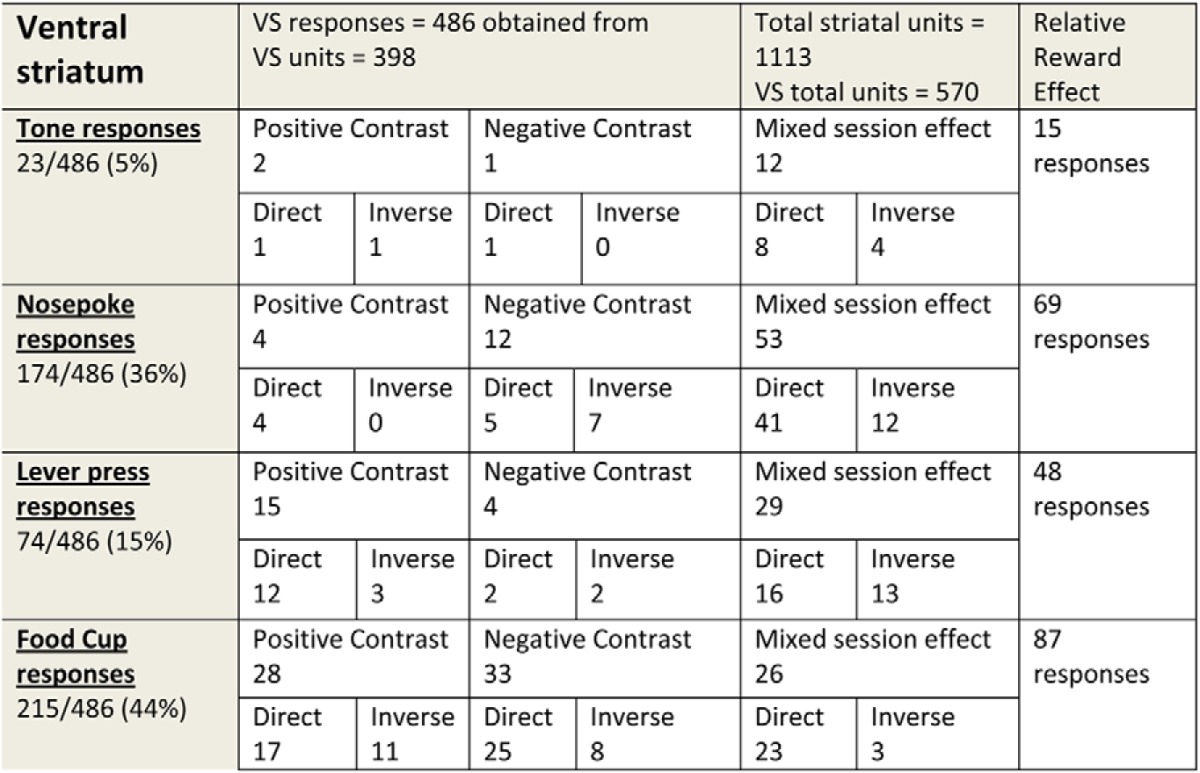
Single units and responses from ventral striatum

Single units and responses from the VS classified by event type and divided by one of the three relative reward effects: positive contrast, negative contrast, or mixed session. See the text for the definition for each of these relative reward effects. Each of these subtypes of responses is divided by the direction of activity change related to reward magnitude.

In the following sections, we will present the details of the responses found to emit activity that fit into the relative reward categories of positive-contrast, negative-contrast, or mixed-session effects. A total of 383 responses from both DS and VS demonstrated one of the forms of relative reward effect. A majority of these relative reward responses were from ventral striatum (239 vs 144 responses; χ^2^ = 7.61, *p* = 0.01) with the slower-firing putative MSN neuron profile [333 of 383 responses with an average baseline firing rate of 2.7 impulses (imp)/s and an average peak-to-valley waveform height of 766 ± 110 µs and average width of 244 ± 33 µs], while a smaller proportion showed faster spiking with smaller, narrower waveforms (50 of 383 responses with an average baseline firing rate of 12 imp/s; mean waveform height, 254 ± 55 µs; width, 99 ± 8.1 µs). For responses emitting direct-excitatory profiles, we found an average of 3.27 ± 1.55 imp/s with a range between 0.48 and 7.92 imp/s. For the inverse subgroup of responses, we found an average of 8.54 ± 2.53 imp/s with a range between 2.99 and 22.10 imp/s.

### Neural activity with positive-contrast effects

#### Dorsal striatum

**Figure 4.**
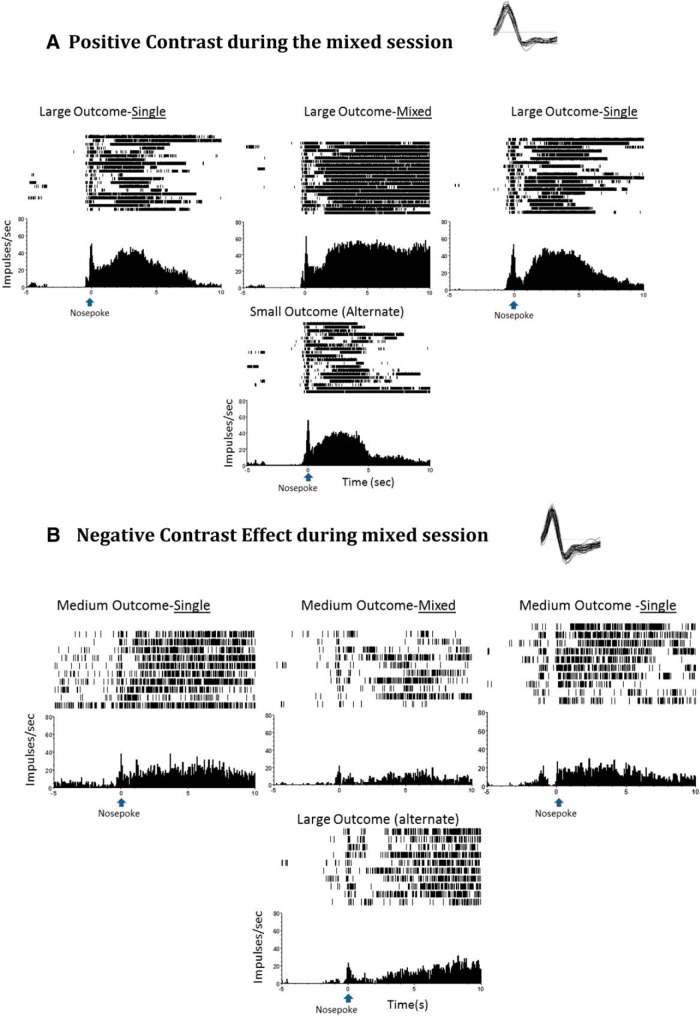
Perievent raster examples of neural activity and incentive relativity. ***A***, Positive-contrast unit: activity from the ventral striatum following the nosepoke response during the sessions, with the top panel showing the response to the larger outcome during the single, mixed, and single sessions. Activity significantly increases to the large (four pellets) outcome in the mixed session when the alternate is the small outcome (one pellet). ***B***, Negative-contrast unit: nosepoke-related unit from the dorsal striatum is significantly active during and several seconds after the nosepoke response. The top panel shows the responses to the medium-sized outcome (two pellets) during the single, mixed, and single sessions. There is significantly reduced activity for the medium outcome in the mixed session when the alternate is the larger outcome (four pellets), signifying a negative contrast effect.

**Figure 5.**
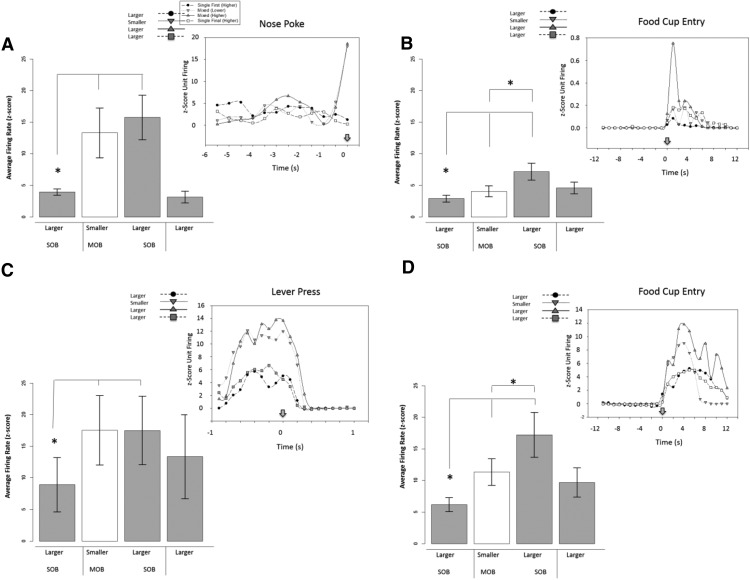
Average neural activity surrounding task-related time points in the positive contrast sessions. Bar graphs represent the average activity change during session exposure, and the insert (top right) provides a depiction of activity changes over time referenced to a task event. In this figure, responses show significantly elevated firing rates in response to larger reward trial in mixed-outcome versus single-outcome condition during positive contrast session types. ***A***, Dorsal striatum nosepoke, 7 responses. ***B***, Dorsal striatum food cup entry, 14 responses. ***C***, Ventral striatum lever-press, 12 responses. ***D***, Ventral striatum food cup entry, 17 responses. Symbols designate significance, as follows: *p < 0.033; &cenveo_unknown_entity_wingdings_0075;*p* < 0.06 (marginal).

**Figure 6.**
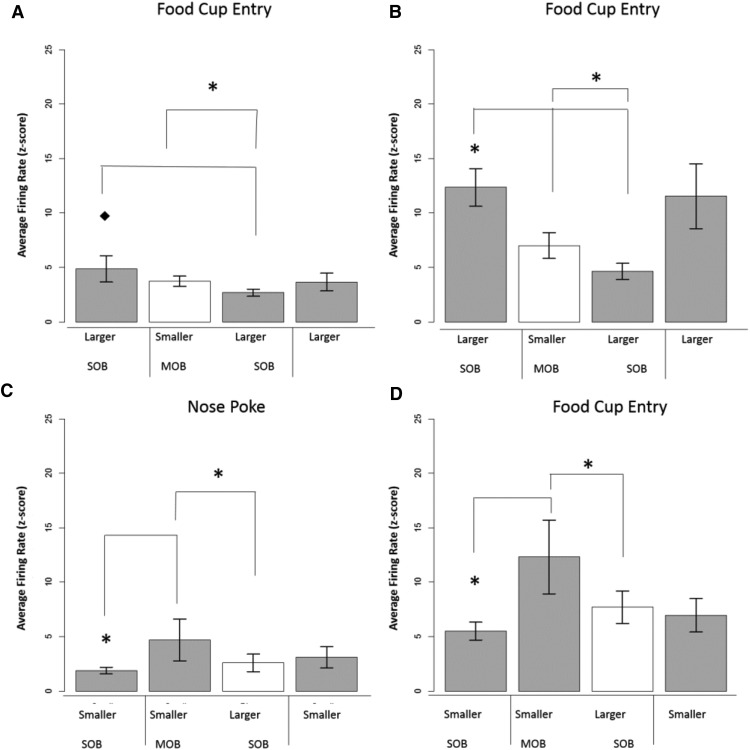
***A–D***, Excitatory inverse unit activity surrounding task-related time points in positive-contrast (***A***, ***B***) and negative-contrast (***C***, ***D***) session types. In this figure, units show an inverse contrast in the mixed-outcome versus single-outcome blocks. Specifically, units during positive contrast show a reduction in firing rates during the mixed-outcome block, while units during negative contrast show an increase in firing rates during the mixed-outcome block. ***A***, Dorsal striatum food cup entry, 10 responses. ***B***, Ventral striatum food cup entry positive contrast, 11 responses. ***C***, Ventral striatum nose poke, 7 responses. ***D***, Ventral striatum food cup entry negative contrast, 8 responses.

We obtained 33 responses showing positive-contrast effects with all of these having significant activity increases for the larger reward during the mixed-outcome block (MOB) compared to the single-outcome block (SOB; Figs. 4B, 5). For the nosepoke response, there were seven responses (six excitatory-direct with waveforms that fit the medium spiny neuron profile; main effect: χ^2^(3) = 11.000, *p* = 0.012; larger-outcome MOB vs SOB: *z* = −2.201, *p* = 0.028). The smaller outcome also showed higher activity during the mixed session (Fig. 5A; MOB vs SOB: *z* = −2.201, *p* = 0.028). This result supports the lack of discrimination between the two outcomes during the trial block with intermixed outcomes. Positive-contrast effects were obtained for two responses following the lever-press in DS (χ^2^(3) = 20.121, *p* = 0.001; larger-outcome MOB vs SOB: *z* = −4.0, *p* = 0.001; smaller vs larger MOB: *z* = −3.327, *p* = 0.002). Twenty-four responses showed positive-contrast effects at the food cup retrieval (Figs. 5B, 6A; main effect: χ^2^(3) = 16.338, *p* = 0.001; larger outcome MOB vs SOB: *z* = −3.296, *p* = 0.001; smaller vs larger MOB: *z* = −3.107, *p* = 0.002). This positive-contrast effect was accompanied by reward discrimination between the two outcomes (smaller vs larger MOB: *z* = −3.107, *p* = 0.002). Nearly half of these responses were direct responding (*n* = 14), and all had firing rates and spike waveform widths to fit the medium spiny neuron subtype (*n* = 24).

#### Ventral striatum

There were significant positive-contrast-like activations after tone onset (*n* = 2; main effect, χ^2^(3) = 23.0, *p* = 0.000; larger outcome: MOB vs SOB *z* = −3.12, *p* = 0.002; and smaller outcome MOB vs SOB, *z* = −3.11, *p* = 0.002) and during the nosepoke (*n* = 4 responses; main effect, χ^2^(3) = 17.100, *p* = 0.001; larger outcome MOB vs SOB, *z* = −2.033, *p* = 0.003; and smaller outcome MOB vs SOB, *z* = −3.139, *p* = 0.001). There were responses linked to the lever-press during positive contrast (Fig. 5C; *n* = 15; main effect, χ^2^(3) = 18.200, *p* = 0.000; larger outcome MOB vs SOB, *z* = −3.059, *p* = 0.002; and smaller outcome MOB vs SOB, *z* = −3.059, *p* = 0.002). Twelve of these fit the direct response and MSN profile of activity. At the time of food pellet retrieval, 28 responses showed positive-contrast-like activation, with 17 responses showing direct responsive activation (Fig. 5D; main effect, χ^2^(3) = 21.494, *p* = 0.0001; larger outcome MOB vs SOB, *z* = −3.621, *p* = 0.0001). These responses showed significant discrimination during the mixed session (*z* = −3.574, *p* = 0.000). The other 11 responses were inverse encoding (Fig. 6B; main effect SOB vs MOB, χ^2^(3) = 17.945, *p* = 0.000). These responses showed significantly lower firing rates during the higher-reward trials in the mixed-outcome block when compared with the initial single-outcome block (*z* = −2.934, *p* = 0.003). These responses also showed a significant inverse discrimination effect, producing significantly lower firing rates for higher-reward trials when compared with lower-reward trials (*z* = −2.934, *p* = 0.003).

### Neural activity with negative-contrast effects

#### Dorsal striatum

There were few significant negative-contrast effects on neural activity at the tone (*n* = 1; main effect, χ^2^(3) = 21.0, *p* = 0.000; smaller MOB vs smaller SOB, *z* = −2.9, *p* = 0.001; smaller vs larger MOB, *z* = −2.162, *p* = 0.020), nosepoke (*n* = 1; main effect, χ^2^(3) = 13.33, *p* = 0.015; smaller MOB vs smaller SOB, *z* = −3.380, *p* = 0.001; smaller vs larger MOB, *z* = −2.32, *p* = 0.039), and lever-press (*n* = 2; main effect, χ^2^(3) = 14.12, *p* = 0.011; smaller MOB vs smaller SOB, *z* = −3.1, *p* = 0.001; smaller vs larger MOB, *z* = −2.072, *p* = 0.039). There was a main direct coding effect at the food cup retrieval (18 responses showed a significant RRE; main effect, χ^2^(3) = 11.123, *p* = 0.011; smaller MOB vs smaller SOB, *z* = −3.180, *p* = 0.001; smaller vs larger MOB, *z* = −2.062, *p* = 0.039). These findings all demonstrate that the neural signals after food delivery were decreased during the mixed session compared with the same outcome retrieved during the single-outcome sessions. Activity consistently showed discrimination between outcomes as well between the smaller and larger reward magnitudes.

#### Ventral striatum

There was 1 tone-related response (lower response to the tone during the mixed session during reward outcome trials; *z* = −2.00, *p* = 0.04) and 12 responses at the nosepoke that showed a negative-contrast effect. Five of these nosepoke responses significantly decreased their firing rates in response to the lower reward trials in the mixed-outcome block when compared with the initial single-outcome block (*z* = −2.04, *p* = 0.043). These responses showed significantly lower activity in the higher-reward trials in the mixed-outcome block when compared with lower-reward trials in the initial single-outcome block (*z* = −2.023, *p* = 0.043). The other seven responses displayed an inverse RRE unit activity (Fig. 6C; χ^2^(3) = 9.343, *p* = 0.025) with higher activity for the lower reward in the mixed session. In addition, there were 4 lever-press responses with the negative-contrast profile (main effect, χ^2^(3) = 13.60, *p* = 0.01; smaller MOB vs smaller SOB, *z* = −2.302, *p* = 0.028; smaller vs larger MOB, *z* = −2.211, *p* = 0.02). Finally, there was a total of 33 responses with negative-contrast effects at the food cup retrieval time, with 25 responses displaying the direct response profile (main effect, χ^2^(3) = 21.528, *p* = 0.000; smaller MOB vs smaller SOB, *z* = −4.372, *p* = 0.000; larger MOB vs smaller SOB, *z* = −3.108) and the other 8 responses displaying the inverse profile (Fig. 6D; smaller MOB vs smaller SOB, χ^2^(3) = 11.800, *p* = 0.018; larger MOB vs smaller SOB, *z* = −2.201, *p* = 0.028).

### Microanalysis of incentive contrast: disparity, bidirectional, and trial-by-trial effects

#### Outcome disparity effects on neural activity and reward relativity

**Figure 7.**
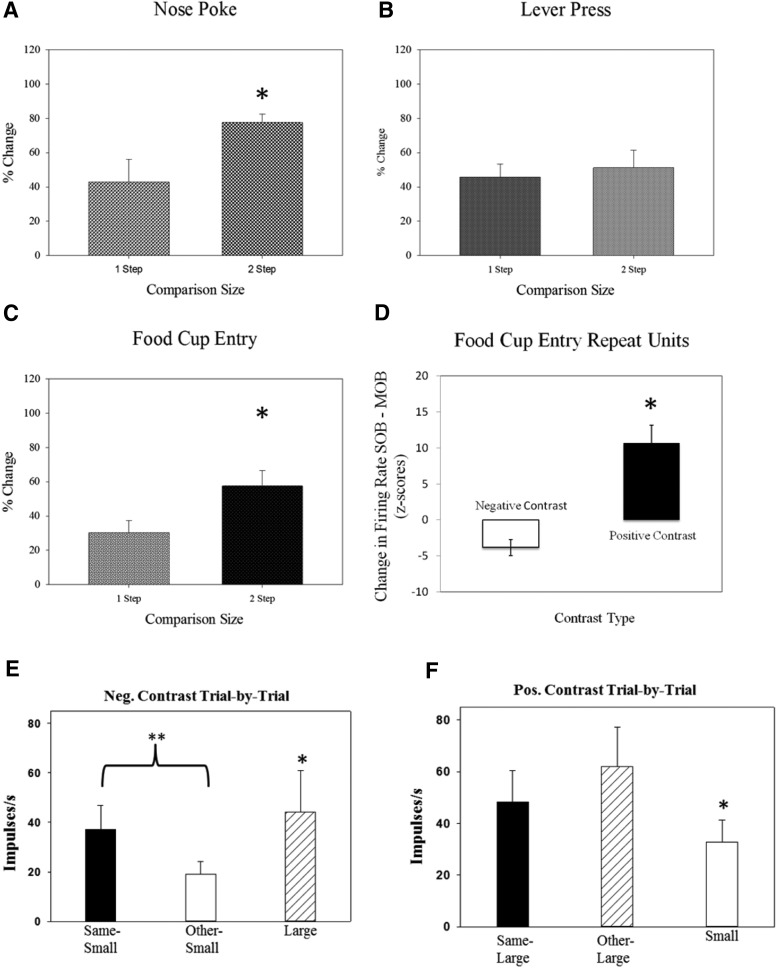
Microanalysis of incentive contrast. Degree of change analysis and striatal activity. One step comparison = RRE percentage change during session types with a small vs medium or medium vs large comparison. Two-step comparison: RRE percentage change during session types with big vs small comparison. *p < 0.033. ***A***, Nosepoke: there was a larger contrast effect in firing rates during two-step vs one-step comparisons surrounding the nose poke response. ***B***, Lever-press: activity for the lever-press response did not show sensitivity to reward disparity, but did show similar levels of response to both one-step and two-step levels of change in reward magnitude. ***C***, Food cup entry: there was a larger contrast effect in firing rates during two-step vs one-step comparisons surrounding the food cup entry response. ***D***, Food cup entry repeat units: firing rates of units that were active surrounding food cup entry in positive- and negative-contrast sessions showed a greater contrast effect in positive-contrast session trials. ***E***, Trial-by-trial analysis and negative contrast: activity was measured post-food cup for trials categorized by the preceding outcome. For the negative contrast comparison, we examined the small-large sessions and compared trials for the small reward preceded by the same outcome (same-small) to trials for small-outcome trials preceded by large-outcome trials (other-small). We found significantly reduced activity in the same-other trials supporting a negative contrast effect based on trial type. ***F***, Trial-by-trial analysis and positive contrast: For positive contrast, we examined large-outcome trials in the single session by comparing those large-outcome trials that were preceded by the same outcome (same-large) to large outcome trials preceded by small outcome (other-large). We found an effect of trial type, but it was nonsignificant between the two large-outcome trial types. **p < 0.01 and *p < 0.05 for all comparisons.

Comparison size was categorized as a one-step (small vs medium; medium vs large) or two-step (small vs large) change in outcome magnitude. One-step comparisons are relatively smaller than two-step comparisons, and it is possible that the size of the comparison has a direct impact on the intensity of the single-unit response-relative reward effect. Results showed that, indeed, this was the case. In the nose poke and food cup entry task-related time points, the size of the activity change was relatively larger during two-step versus one-step comparisons (Fig. 7A–C). This evidence further supports the idea that disparity during relative reward effects is a parametric process with a degree of scaling that is sensitive to a relative context as well as degree of disparity among reward values.

#### Units demonstrating both positive and negative contrast

Responses that were active in more than one daily session were analyzed. These responses were obtained in both positive- and negative-contrast session types. The same degree of change analysis described in the previous step analysis section was performed on these repeat units. This was done to examine whether repeat units were more sensitive to positive or negative comparison types. There was a total of 10 repeat responses for the food cup entry task-related time points. These units were sensitive to both negative and positive contrast, but demonstrated a greater contrast effect in positive-contrast versus negative-contrast sessions (Fig. 7D). There were two units that showed a repeat relative reward effect during the lever-press task-related time point, but this sample size was too small for statistical analysis.

#### Trial-by-trial analysis of incentive contrast

In order to examine dynamic properties of contrast at a finer level, we divided mixed sessions into the following two trial types: same and other. “Same” trials were outcomes preceded by the same reward while “other” trials were preceded by the alternate. We then divided the other category into the following two subsets: small preceded by large and large preceded by small. We used 21 responses from five animals, and all were responsive around the food cup (4 s time period following food cup response, 60 ms bins for each unit). This analysis enables comparisons between individual sets of trials for either large or small outcomes conditional upon the preceding trial type. We used a nonparametric statistical test (Wilcoxon signed-rank test) to examine the differences between the neural activations during these specific trial types because of the heterogeneity of firing rates and variance among the responses sampled (firing rate range for responses, 19–200 spikes/s; variability range, 23–76 spikes/s).

For negative contrast between trials, we compared responses from trials for the smaller outcome preceded by the same trials to responses found during the smaller-outcome trials preceded by large-outcome trials all in the mixed-outcome session. We found a significant decrease in activity in the small-reward trials following large outcomes compared with small-outcome trials following a small reward (*W* = 18, *p* < 0.01; Fig. 7E). Additionally, within the mixed session, we found a significant difference between the large and small trials (*W* = 18, *p* < 0.01). For positive contrast, we compared responses from the large-reward trials following small outcomes with responses from the large-outcome trials following other large-outcome trials. Despite an increase in firing rate (Fig. 7F), the difference was nonsignificant, with high levels of variability in the set of responses. There was a significant difference between the large and small trial types within these mixed sessions as well. These results highlight the rapid nature (trial-by-trial) of the plasticity for relative effects on neural activity. The striatum may not “act” on these transient shifts in neural activity but store them to be used for future reference in developing comparisons and integrating information to form comparisons built upon more diverse information (i.e., internal state shifts related to satiety).

### Neural activity with “mixed-session” effects

#### Dorsal striatum

**Figure 8.**
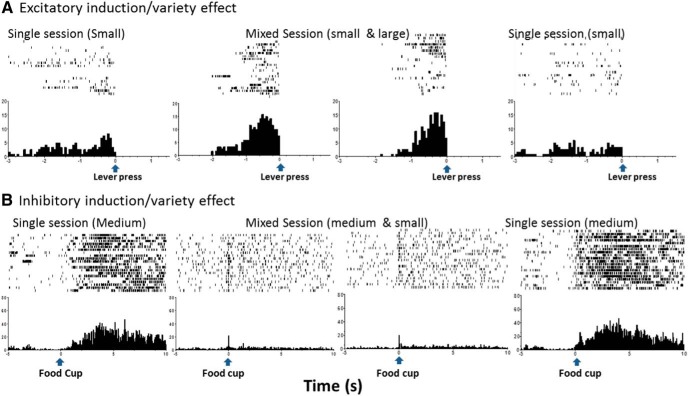
Perievent raster examples of relative reward effects that resemble positive induction or variety influences. ***A***, Activity increase during mixed session: neural activity from the dorsal striatal MSN is timelocked to lever-press and significantly increases for both outcomes during the mixed session (small and large outcomes alternating). ***B***, Activity decrease during the mixed session: neural activity from a ventral striatal unit MSN is related to the food cup entry and is significantly reduced in the mixed session (medium and small outcomes) and returns to premixed session levels in the final single session.

**Figure 9.**
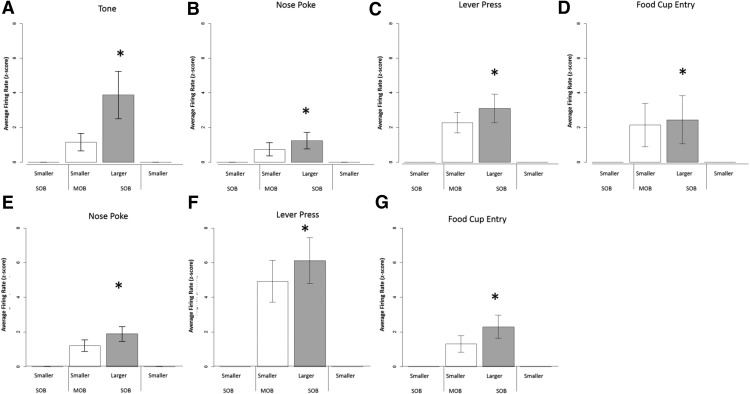
***A–G***, Reward discrimination found in direct coding mixed-session units during contrast sessions in the dorsal (***A–D***) and ventral (***E–G***) striatum. These units showed faster firing rates in response to larger vs smaller tones in the mixed-outcome block only. ***A***, Tone, 9 responses. ***B***, Nosepoke, 7 responses. ***C***, Lever-press, 9 responses. ***D***, Food cup entry, 13 responses. ***E***, Nose poke, 15 responses. ***F***, Lever-press, 12 responses. ***G***, Food cup entry, 19 responses.

Mixed-session effects were placed into the following two distinct categories: (1) neural responses that changed in the mixed session in a similar intensity and direction for both outcomes in an opposite manner to that predicted by incentive-contrast effects; and (2) significant neural responses of event-related responses obtained only during the middle mixed session. For the first category, these similar responses (*p* > 0.05) during the mixed session for the larger and smaller outcomes were observed at the nosepoke response (*n* = 6 direct responses from positive-contrast sessions) and during the lever-press (eight direct responses; four from positive-contrast sessions; Fig. 8A). For the second category, responses significantly discriminated between the two outcomes during the mixed session (Fig. 9A–D). During positive-contrast sessions, five nosepoke task-related responses showed a significant discrimination effect between outcomes (*z* = −2.123, *p* = 0.03). During negative-contrast sessions, nine tone (Fig. 9A; *z* = −2.213, *p* = 0.033), seven nosepoke (Fig. 9B; *z* = −2.07, *p* = 0.02), nine lever-press (Fig. 9C; *z* = −2.201, *p* = 0.028), and 13 food cup entry (Fig. 9D; *z* = −2.803, *p* = 0.005) task-related responses showed a significant discrimination between the larger and smaller reward outcomes. Negative-contrast sessions also revealed inverse coding responses in the dorsal striatum. Four of these responses following tone onset, 15 responses surrounding the nose poke (*z* = −2.667, *p* = 0.008), and 13 responses surrounding lever-press (*z* = −3.059, *p* = 0.002) showed inverse reward discrimination.

#### Ventral striatum

VS response activity was categorized in the same mixed-session profiles. Neural activations showing similar significant shifts in activity during the mixed session were found linked to the nosepoke (*n* = 18; all direct coding from positive contrast) and the food cup response (*n* = 7 with three inverse and from negative-contrast sessions; Fig. 8B). The second form of mixed-session profile included responses only during the mixed session with eight nosepoke responses (*z* = −2.223, *p* = 0.03) and eight tone responses showed a significant discrimination effect (*z* = −2.521, *p* = 0.012) during the positive-contrast sessions. During negative-contrast sessions, 15 nosepoke (Fig. 9E; *z* = −3.408, *p* = 0.000), 12 lever-press (Fig. 9F, *z* = −2.521, *p* = 0.012), and 19 food cup entry (Fig. 9G; *z* = −3.296, *p* = 0.001) task-related responses showed significant reward discrimination. Negative-contrast sessions also revealed inverse coding within the ventral striatum. Four post-tone stimuli, 12 responses surrounding the nosepoke (*z* = −2.667, *p* = 0.008), and 17 responses surrounding the lever-press (Fig. 8; *z* = −3.181, *p* = 0.001) showed inverse reward discrimination.

## Discussion

The present findings support the idea that striatal reward processing is dynamic and reflects changes in the incentive value of outcomes over time. The results are unique in revealing the rapid nature of this dynamic process of reward revaluation in striatum and support shifts in valuation similar to an “instant transformation” of outcome value from highly aversive to appetitive (Tinklepaugh, 1928; Berridge and Cromwell, 1990; Robinson and Berridge, 2013). This updating can occur parametrically within both positive and negative valences, and can integrate both previously learned values as a reference with current experience. One important difference between the present work and the majority of other work on striatal neural plasticity is that learning and probability were kept constant throughout the recording periods. Neural computations in the present work were independent of changes in risk or predictability (Ma and Jazayeri, 2014). Instead of value updating that is reliant upon prediction error signals, the dynamic value arose out of an established hierarchy of outcomes anchored by a baseline or absolute value (Seymour and McClure, 2008). Another important piece of the results is the uncovering of striatal activity representing different levels of outcome processing simultaneously. Future work should examine how these different levels interact with possible candidate interactions, including hierarchical levels of function (nested interaction) or independent parallel value coding. In the former idea of linked interactions, one possibility is that different specific levels of encoding would be guided by a general level of context. For example, if the numbers of alternatives increase along with alternative variety, then the weighting of specific outcomes could change because the probability of being exposed to any one outcome could significantly decrease. Other alternatives could exist, as well including reciprocal interactions with equal weighting between levels or even a dynamic relationship with value coding shifting conditional to environment and experience. These relationships between different levels of outcome processing would allow for flexibility depending upon intrinsic factors of hunger or satiety and external factors of food scarcity or abundance. For example, during sparse food availability, relative reward effects may be dampened, and behavioral work in psychology has suggested such an interaction between valuation and internal-state modulation (Sears et al., 2010; Bissonette et al., 2013).

The idea of differential value coding has been proposed to explain the functional nature of basal ganglia activity (Hikosaka et al., 2014) and includes roles for distinct neural ensembles that encode stable versus flexible value representations (Silberberg and Bolam, 2015). This flexible encoding broadens from single events, actions, or outcomes to include molar aspects of the outcome context. One form of flexible value coding emphasizes contrast and forms a set of comparisons that includes the following: (1) the distinction between outcome alternatives in the present; and (2) the distinction between identical outcomes in the past versus the present experience. We found that responses in both dorsal and ventral striatum were sensitive to positive and negative contrast. These activations were linked to events preceding the outcome and during acquisition of the food outcome. A more detailed analysis of these signals showed that the relative comparisons were enhanced when examined with trial-by-trial sequences of different outcomes. This more molecular analysis, or microanalysis, of contrast could be extended to examine sequential dependencies regulating the reward comparisons during both simple and more complex choice situations (Silberberg et al., 1978). These dynamic striatal activations could combine with stable representations. Stable activity would be linked to events and outcomes, and not significantly change within the timescale under study. These two different types of representations could produce accurate value updating, and, in the short term, they would be used to compute an immediate value temporarily stored.

Striatal activity influenced by relative reward was linked to appetitive behavior during reward acquisition. This supports work using nucleus accumbens (NAcc) lesions that found selective effects on instrumental behavior and not consummatory actions (Lesczuk and Flaherty, 2000). Input from prefrontal cortical regions could guide these relative reward effects and provide access to value representations. Orbitofrontal cortex lesions interrupt the updating of the incentive value of cues that have been devalued (Gallagher et al., 1999), and neurons in the orbitofrontal cortex encode relative reward value (Tremblay and Schultz, 1999). This latter work showed that OFC neurons alter activity by completely shifting to the cues and outcomes that represent the more preferred reward outcome. This particular activity was found for outcome expectancy-related activations, and these responses could combine with activity in the striatum to produce changes during reward acquisition.

By our criteria, we subdivided responses using the changes in activity during the series of three sessions containing either a single-outcome or mixed outcomes into different forms of relative reward effect. A contrast relative reward effect includes a significant difference in the predicted direction based on the contrast valence (greater shift for positive and reduced activity change for negative) between the sessions for a single-reward outcome experience. These specific functions could rely on striatal communication with other subcortical regions, such as the hippocampus, amygdala, and brainstem circuits (Liang et al., 2012; Nyland et al., 2012). Lesions to the hippocampus alter shifts in instrumental responses during negative contrast (Liao and Chuang, 2003). Hippocampal function has been linked to reward processing and outcome valuation (Bett et al., 2015; Kanoski and Grill, 2015). Basolateral amygdala (BLA) muscimol infusion interrupted the ability for rats to express previously learned stimulus–outcome associations in a differential outcome procedure (Savage et al., 2007). Lesions of the BLA interfere with the ability of animals to update the values of rewards (Corbit and Balleine, 2005; Haney et al., 2010). Specifically, animals with basolateral amygdala lesions lever-press normally to a cue that has been associated with reward delivery even after the reward has been devalued with lithium chloride (LiCl; Corbit and Balleine, 2005). Additionally, lesions of the basolateral amygdala interrupt extinction when that cue is presented without the expected reward (Lindgren et al., 2003). Inactivation of BLA results in a dramatic reduction in firing rates of NAcc neurons during the processing of rewarded cues and stimulation resulted in significant increases in neuronal firing of neurons in the NAcc (Ambroggi et al., 2008). Other work using standard incentive-contrast paradigms has shown brainstem regions can contain sufficient neural operations to produce incentive contrast (Grigson, 1997). This work focused on consummatory contrast with upshifts and downshifts in lick rates (Grigson et al., 1993, 1994). The striatal region provides an intersection point for cortical, subcortical, and lower brainstem regions to interact, and a possible location for output to be organized into effective motivated behavior (Grill and Kaplan, 2002).

A second major form of relative effect was observed that excludes contrast effects because the definition for “mixed-session” effects includes responses that either do not have a significant activation during the single session (mixed-session selective) or have an activity shift during the mixed session in the opposite direction to the one predicted by incentive contrast (greater activity for negative contrast and reduced activity for positive contrast). These forms of mixed-session effects could be viewed as highly dependent upon the context of the mixed session having multiple outcomes. Context effects can arise from general location (Mizumori, 2013), emotion (Binkley et al., 2014; Moorman and Aston-Jones, 2014), and temporal processing (Genovesio et al., 2016). In most cases, context effects arise from a global set of information comprised of many details but organized as a whole (Tversky and Kahneman, 1981). Previous work has focused upon probability or risk as a context cue within the environment (Aw et al., 2012). Neural activity can dissociate context-dependent encoding from specific reward value (Schultz et al., 2011; Schultz, 2012). Dissociations could arise when conflicts between specific and context value occur.

We have used the term “variety” to describe context-dependent activity and found that this form of activity did appear selectively during the mixed-outcome sessions (Webber et al., 2015). Most dramatically, the activity appears as uniform responses that are selective for the mixed session. On average, the activity did discriminate between the outcomes during the session. This indicates that a general context signal combines with information about the individual outcomes. Variety effects on behavior are complex and often lead to enhanced or prolonged behavioral responding (Rolls et al., 1983; Treit et al., 1983). The neural basis for variety effects has been studied mainly as a reduction in sensory-specific satiety signals in the orbitofrontal cortex (OFC), basal ganglia, and hypothalamus (Rolls et al., 1986; Rolls, 2000). These sensory-specific satiety signals have been primarily linked to specific food outcomes. The process is akin to a dishabituation effect in that a new food substance removes the habituation toward a pre-exposed food item and reignites the motivation for consumption (Bouton et al., 2013; Thraikill et al., 2015). Our signals could be working in this manner, and it would support the result that these activations that dishabituate are robust solely in the mixed session with exposure to the alternatives is necessary for the neural effect to take place.

Another form of mixed-session effect response did demonstrate activity in both single and mixed sessions but did not follow the incentive-contrast predictions. Induction has been offered as a label to describe behavioral responses during contrast that, instead of becoming more distinct, actually become more similar (Weatherly et al., 2001). In most forms of induction, behavioral responses generalize to distinct outcomes and discrimination breaks down (Weatherly et al., 2005, 2006). Behaviorally animals may emit generalized responses as outcomes become more similar or as anticipation for one outcome leads to an invigoration of responding for all outcomes. We found mixed-session effects opposite to contrast that do resemble these behavioral effects. Neural activity was greater for the lower value outcome when it was paired with a higher valued outcome. In this case, the generalization could lead to an invigoration of responding to all outcomes that rises to the level of the most valued outcome with the set (Webber et al., 2015). Work showing generalization reflected by neural activity is critical for learning and cognitive associative networks to operate for adaptive emotion and behavior (Engineer et al., 2013; Ghosh and Chattarji, 2015). Neural responses could reflect a merging process despite disparate absolute values.

The striatum is functionally heterogeneous with dorsal and ventral functional specialization (Burton et al., 2015; Tellez et al., 2016), as well as subregions within dorsal and ventral areas having distinct roles (Cromwell and Berridge, 1996; Kimchi and Laubach, 2009). For example, Kimchi and Laubach (2009) found that single units in dorsal striatum were sensitive to a response bias in lever-pressing for liquid reward. Related to the present findings, activity was dynamically influenced by actions emitted in the previous trial. Other work (Smith and Graybiel, 2016) has recently highlighted the plasticity of striatal activity focusing on dorsal subregions. The present work found relative outcome encoding in both dorsal and ventral subregions. Greater activity was found in the nucleus accumbens, especially for the responses surrounding the reward acquisition. The higher levels of outcome-related activations in ventral striatum is well documented (Schultz et al., 1993), and our findings support the notion that the striatum is divided into dorsal–sensorimotor and ventral–outcome encoding subareas. We did find significant differences between the dorsal or ventral subregions, with the ventral subregion containing significantly greater numbers of responses showing relative reward effects. A novel approach could include an investigation into these different levels of outcome processing and how particular subregions and inputs are more involved in general outcome information processing versus specific, individual outcome information. More effort on this issue could involve examining action value at different levels of operation or as the context of action varies (e.g., from forced to free choice; Premack, 1962; Ricker et al., 2016a). Recent data show that striatum is more crucially involved in components of choice within a free environment as opposed to a more forced or sequential choice environment (Ricker et al., 2016b). Future work could examine functional heterogeneity of striatal reward processing by varying these levels of both outcome and action complexity.

Significant deficits in choice behavior and decision-making related to reward and outcome acquisition are found in mental illness (Green et al., 2015), and, interestingly, these can be observed when “absolute value” coding remains intact. In particular, for schizophrenia, several research teams point to a significant problem in reward valuation (Strauss et al., 2014). When values shift over time, patients with schizophrenia have problems in shifting behavior accordingly and in tracking value changes as alternatives or outcome parameters shift. The functional neuroimaging data suggest that the striatum activity could be altered in these individuals. For example, less activation in ventral striatum was found when patients showed altered delayed discounting (Gold et al., 2013). In this paradigm, the relative valuation depends upon the wait time until reward acquisition, with lower value the longer one waits.

Alterations in reward comparison could occur in addiction and result from inabilities to update value adaptively (Grigson, 2000; Schultz, 2011; Jentsch et al., 2014). One basic consequence of drug exposure could be devaluation of nondrug outcomes. These changes could occur rapidly and have an impact on reward comparisons in general. It requires only one saccharine–morphine pairing to reduce the motivation to consume saccharine (Grigson and Hajnal, 2007), suggesting that drugs of abuse produce powerful contrast effects over naturally rewarding stimuli. Microdialysis revealed that the single saccharine–morphine pairing led to a significant decrease in dopamine levels found in the striatum during saccharine exposure (Grigson and Hajnal, 2007), showing that these drug-induced contrast effects cause changes in how the brain processes natural rewards versus how it processes drug rewards. Furthermore, it has been found that dopamine levels are sensitive to relative reward alterations (Genn et al., 2004) and that animals under d-amphetamine withdrawal produce enhanced negative-contrast effects for natural rewards (Barr and Phillips, 2002). Understanding the substrates of relative reward processing in typical cases or cases related to mental illness could provide insights into the basic science of motivation and the study of related behavioral impairments.

## Synthesis

The decision was a result of the Reviewing Editor Philippe Tobler and the peer reviewers coming together and discussing their recommendations until a consensus was reached. A fact-based synthesis statement explaining their decision and outlining what is needed to prepare a revision is listed below.

This revised manuscript examines the activity of rat striatal neurons during instrumental behavior, with a focus on identifying neural reward-related responses related to incentive contrast effects. Incentive contrast effects describe behavior in which responses to a given reward depends on other rewards offered in the past or as an alternative; while long-established in the behavioral literature, the neural basis of these effects are unknown. The authors use a relative reward task for which they have previously reported behavior; in this task, tone-reward contingencies are either presented alone in a block or in a paired combination across individual trials, enabling examination of how neurons respond to a given reward magnitude as a function of the larger reward environment (context, or contrast condition).

It is clear that the authors have made a good effort in revising the manuscript to streamline the figures, clarifying details of experimental protocols and physiological analyses, and address concerns regarding the significance of the findings. However, the current revised manuscript still has a number of issues which need to be addressed. The main concern is whether the neural data show convincing enough relative reward effects to allow definitive conclusions about the role of striatum in incentive contrast effects.

Major points

1) Significance of the observed neural effects. The revisions to the manuscript, particularly in extending the technical description in the methods and reformulating the tables, have improved the readability and clarity of the manuscript. My one remaining concern is the significance of the observed neural relative reward effects given the low percentage of neurons demonstrating these effects. While the percentage of striatal neurons active in the task seems comparable to other studies (11-39%, depending on the area and phase of task), the proportion of neurons demonstrating RRE effects are a substantially smaller fraction. For example, one of the largest proportions is in positive contrast sessions at the food cup, at 7.3% (24/328), with many of the proportions smaller (0.61% [2/328] at the lever press for positive contrast).

The authors report that the total number of neurons showing RRE effects is ∼20% ("274 (20 of total units)", line 393), but it's not clear how this number is derived and it seems like an overestimation. If there are a total of 1400 recorded neurons (if "units" at any point is meant to refer to responses, the authors should use "responses" instead of "units"), but each of them were examined in different task epochs, adding up the number of units across different epochs is not appropriate as they may be the same neurons responsive in different conditions. Similarly, the mixed session effects were examined in the same trials as positive/negative contrast effects; you can't add these together to report a total number of neurons with RRE effects. Can the authors clear this up?

In addition, the multiple comparisons correction used addresses the issue of examining multiple bins, but does not correct for testing for RRE effects across multiple neurons: in a large sample of neurons, a small number of neurons coding differences in activity along a chosen dimension would be expected by chance. Most of the statistics reported for the RRE effects (e.g. "For the nosepoke response, there were 6 units all excitatory-direct with waveforms that fit the medium spiny neuron profile (main effect: X2(3) = 11.000, p = .012; Larger outcome MOB vs. SOB: Z = -2.201, p = .028)", lines 400-402)") seem to have been performed on neurons already identified to show the effect. If this is true, then these analyses don't address the significance of the coding across the population. Can the authors address this point?

In the end, I think the authors can reasonably argue that the data stands by itself, and that even a small percentage of neurons demonstrating RRE links striatal activity to the behavioral effects. However, at the minimum, the authors need to correct how they are determining the total number of RRE-coding neurons.

Other points

1) Hypothesis. The addition of hypotheses at the introduction is a helpful addition to framing the rest of the manuscript. However, the specific hypothesis that "the "neural signal related to relative valuation will be more prominent in the ventral striatum (VS) compared to the dorsal striatum (DS) especially surrounding reward delivery and acquisition" (lines 115-117) comes out of the blue, and doesn't follow from any of the preceding introductory paragraphs. From a reader's perspective, why is this the authors' hypothesis? At the minimum, the authors should include a brief discussion of the known differences in VS vs. DS information processing to support this particular hypothesis.

2) Trial-by-trial analyses. The presentation of the trial-by-trial effects is confusing, specifically in regards to how the analyses were done. Presumably, the authors wish to compare, for example, responses to a small reward in a mixed small/large block when following a large preceding reward versus a small preceding reward. This would provide a trial-by-trial analysis of dynamic changes in incentive contrast coding within a block. This within mixed-block analysis matches the description on lines 467-470. However, the descriptions in the subsequent paragraph (and the Fig 7E legend) suggest that the comparison is *between different blocks*: "For negative contrast between trials, we compared small outcome trials from the mixed session to the small outcome trials in the single session" (lines 477-478). Examining this across blocks seems to replicate the general contrast effects already presented (e.g. Figs 5 and 6).

3) Presentation of mixed session neural effects. The presentation of the mixed session effects are also a little unclear. First, Figure 8 is never clearly discussed in the text, and a few sentences (preceding the text at line 491) describing what the authors intend to examine in the mixed session data would be helpful to the reader. The induction/variety examples shown in Fig. 8 are not discussed in the results, and it is not clear how the examples shown in Fig. 8 relate to the data shown in Fig. 9 (if they do so at all). Second, the results presented in lines 492-507 could be more clearly presented. The authors describe neurons with a "significant discrimination effect" (lines 493, 496, 501, 504) but never state what the neurons are discriminating between: is it between the different rewards in the mixed sessions?

4) Number of recording sessions. The authors have clarified a great deal regarding the electrophysiology and overall task design. One thing that is currently missing that should be included is the number of recording sessions. There were 6 animals and 6 possible SOB-MOB-SOB daily sessions; was each animal run on each session? were sessions repeated in each animal? I suggest the authors include the total number of recording sessions near the initial presentation of neural data (line 368).

5) Dissociation of contrast and variety effects. The introduction presents different ways in which behavior and neural responses can change with context changes. However, the neural analysis does not distinguish between these possibilities. This is particularly relevant for contrast and variety in that some of the neurons presented as contrast neurons may indeed be better characterized as variety neurons. The distinction between different relative effects should also be taken up again in the discussion.

6) Statistics. Across behavioral and neural data analyses I counted 20 instances of tests with the exact same test statistic (Z=-2.201, p=0.28), which seems clearly more than what would be expected by chance.

7) Readability: there are still a number of places with grammatical or spelling errors:

- line 67: "repaid comparisons"

- line 115: "that neural signal related to"

- line 162: "as administered"

- line 173: "orders"

- line 182: "recovery"

- line 271: "binds"

- line 283: "set of analyzes"

- line 455: "RRE is truly relative processes, sensitive to a relative context"

- line 458: "than session"

- lines 571-572: "lesions of the basolateral interrupt"
